# Epidemiological Patterns of Genital Ulcer Disease and Human Immunodeficiency Virus Among Public Clinic Attendees in Mthatha, Eastern Cape, South Africa

**DOI:** 10.3390/diseases13090293

**Published:** 2025-09-05

**Authors:** Thembisa R. Tshaka, Lindiwe M. Faye, Teke R. Apalata, Zizipho Z. A. Mbulawa

**Affiliations:** 1 Department of Laboratory Medicine and Pathology, Faculty of Health Sciences, Walter Sisulu University, Mthatha 5100, South Africa; lfaye@wsu.ac.za (L.M.F.); tapalata@wsu.ac.za (T.R.A.); 2 National Health Laboratory Service, Nelson Mandela Academic Hospital, Mthatha 5100, South Africa

**Keywords:** disease prevalence, testing coverage, sex disparities, age-specific trends, co-occurrence analysis, GUD, public health interventions

## Abstract

Background: Sexually transmitted infections (STIs) are common globally, posing significant public health challenges and financial strain, especially in low- and middle-income countries. Sub-Saharan Africa (SSA) accounts for 40% of global STI prevalence, with South Africa having the highest rates of curable STIs and human immunodeficiency virus (HIV), both of which are closely linked to increasing HIV transmission risk and other STIs. Genital ulcer disease (GUD), primarily caused by HSV-1, HSV-2, and *Treponema pallidum*, and less frequently by *Haemophilus ducreyi*, *Klebsiella granulomatis*, and *Chlamydia trachomatis*, exemplifies the complex interplay of STIs. Methods: This study analyzed GUD and co-infection with HIV, testing patterns, and co-occurrence trends among public clinic attendees in Mthatha, South Africa, to identify demographic, behavioral, and occupational disparities. Results: Sex-specific analysis revealed higher HIV prevalence among female attendees (47.00%) compared to male attendees (22.00%), alongside notable testing gaps and disparities in diseases such as syphilis, genital herpes, and lymphogranuloma venereum (LGV). Age-specific trends indicated the highest HIV prevalence in individuals aged 30–49, with peaks at 66.67% (30–39) and 76.47% (40–49). *Treponema pallidum* and HSV-2 prevalence were most pronounced in younger age groups (<20 and 20–29), while older demographics (50+) exhibited significant diagnostic gaps. Occupation-based analysis highlighted elevated HIV (65.91%) and HSV-2 (19.61%) prevalence among unemployed individuals, reflecting socioeconomic vulnerabilities. Co-occurrence analysis revealed notable overlaps, such as HIV and HSV-2 (6.67%) and *Chlamydia trachomatis* with HSV-1 (5.71%) and HSV-2 (4.76%), driven by shared risk factors. Correlation analysis identified strong links between HSV-1 and *Haemophilus ducreyi* (0.64) and between *Chlamydia trachomatis* and HSV-1 (0.56), underscoring the potential for integrated diagnostic strategies. Conclusion: These findings emphasize the need for targeted public health interventions addressing sex, age, and occupational disparities while improving diagnostic coverage and prevention efforts for co-occurring infections.

## 1. Introduction

Sexually transmitted infections (STIs), among the most common acute infectious conditions globally, pose a significant public health challenge, straining household and national health budgets while adversely affecting individuals’ quality of life despite rarely being life-threatening [1]. Low- and middle-income countries continue to bear a disproportionate burden, with Sub-Saharan Africa (SSA) contributing to 40% of the global prevalence of STIs. Within SSA, South Africa has the highest rates of curable STIs and human immunodeficiency virus (HIV), with the two epidemics closely linked, as STIs significantly heighten the risk of HIV transmission and acquisition [2]. Dhar et al. (2024) define genital ulcer disease (GUD) as ulcerations resulting from STIs, primarily due to genital herpes caused by herpes simplex virus types 1 and 2 (HSV-1 and -2) and syphilis caused by *Treponema pallidum* [3]. Less frequently, it is associated with chancroid caused by *Haemophilus ducreyi*, granuloma inguinale (donovanosis), caused by *Klebsiella granulomatis*, and lymphogranuloma venereum (LGV), caused by *Chlamydia trachomatis* [3].

Understanding disease prevalence and testing patterns is essential for developing targeted public health interventions. This study analyses sex, age, occupation, and co-occurrence trends among clinic attendees, highlighting critical disparities in disease distribution and diagnostic coverage. Sex-specific analysis reveals a higher prevalence of HIV and syphilis among female attendees compared to male attendees, while testing gaps for multiple diseases are more pronounced in female attendees [4]. A study conducted in Zambia also recorded a HIV prevalence among young women aged 15–24, which is significantly higher (5.6%) compared to their male counterparts (1.8%) [5]. Conversely, with syphilis statistics, another study reported that 97.9% of syphilis-positive individuals were male, with 66.3% identifying as men who have sex with men (MSM) [6]. Age-based trends show that HIV and syphilis prevalence peak in middle-aged groups, with younger populations exhibiting elevated rates of HSV-2 and *Chlamydia trachomatis* [4]. Gilbert et al. (2021) found that the median age of individuals with HIV and syphilis co-infection was 38 years, suggesting higher prevalence in middle-aged groups [7]. Older adults face significant testing gaps across multiple conditions, emphasizing the need for improved diagnostic efforts in this demographic.

Occupation-based analysis identifies unemployed individuals as particularly vulnerable, with higher prevalence rates for HIV, syphilis, HSV-1, and HSV-2 compared to employed or student groups, indicating socioeconomic disparities in disease burden [8,9]. Studies have demonstrated that unemployed individuals exhibit higher prevalence rates of STIs, including HIV, syphilis, HSV-1, and HSV-2, compared to their employed or student counterparts. This disparity underscores the influence of socioeconomic status on disease burden [10]. Significant overlaps, such as those between HIV and HSV-2 and those between *Chlamydia trachomatis* and HSV-1/HSV-2, suggest shared risk factors and transmission pathways [11]. The co-infection of HIV and HSV-2 is well-documented, with HSV-2 infection increasing susceptibility to HIV acquisition. Additionally, co-infections involving *Chlamydia trachomatis* and HSV-1/HSV-2 are prevalent, suggesting shared risk factors and transmission routes [12]. Conversely, diseases like syphilis and granuloma inguinale show minimal overlap, reflecting distinct epidemiological profiles [13]. Research indicates minimal overlap between infections like syphilis and HSV-2, reflecting distinct epidemiological profiles and transmission dynamics [10].

This study’s additional analysis extends the original study’s findings of [4] by addressing gaps in knowledge regarding GUD in the Eastern Cape Province of South Africa, particularly in the King Sabata Dalindyebo local municipality. By providing a deeper understanding of epidemiological patterns, the new analysis focuses on sex, age, and occupational disparities in GUD prevalence and co-occurring HIV infection. Furthermore, it emphasizes the relationship between GUD and other STIs such as HIV, genital herpes, and syphilis, offering a detailed examination of co-occurrence trends among public clinic attendees. This provides insights into overlapping infection risks and potential transmission pathways, which were not explored in-depth in the original study. Additionally, the extended analysis highlights critical socioeconomic and demographic insights, identifying specific groups, such as unemployed individuals, who exhibit a higher burden of disease, underscoring the need for targeted public health interventions. By examining disease testing coverage and identifying diagnostic gaps, this analysis supports improved public health strategies aimed at enhancing STI diagnostic efforts, particularly for vulnerable groups like older adults and women. The incorporation of statistical and correlation analysis between different pathogens, such as HSV-1 and *Haemophilus ducreyi*, further emphasizes the importance of integrated diagnostic strategies, providing a more comprehensive understanding of disease co-occurrence patterns. This level of analysis supports more effective resource allocation for GUD and HIV prevention and treatment in the region.

The study was conducted in Mthatha, located in the King Sabata Dalindyebo (KSD) Local Municipality, within the Oliver Reginald (O.R.) Tambo District Municipality of the Eastern Cape Province, South Africa. This region includes both urban and rural settlements and is characterized by high burdens of poverty, limited healthcare infrastructure, and elevated rates of HIV and other STIs. This comprehensive study approach aims to inform future efforts to reduce the burden of GUD and its associated infections, ultimately improving health outcomes in this limited healthcare infrastructure region.

## 2. Materials and Methods

A retrospective cross-sectional analysis was conducted using data from Tshaka et al. (2022) [4], in which data were generated between May 2018 and July 2019 from public clinic attendees in Mthatha, South Africa. All available data were used, which included demographic variables (sex, age, residence, occupation, and education), behavioral factors (substance abuse, number of sexual partners), and laboratory results for the following diseases: HIV, syphilis, genital herpes, lymphogranuloma venereum, chancroid, and granuloma inguinale. Participants were included in the study if they presented with visible, unhealed genital ulcers during routine visits to public CHCs in Mthatha, Eastern Cape, South Africa, and self-reported signs and symptoms of GUD. Eligible participants were required to provide written informed consent to participate in the study. They also underwent a private interview to collect socio-demographic and clinical information, followed by a clinical examination and sample collection. Furthermore, blood and genital ulcer swab samples were collected from each participant and tested for the presence of HIV, HSV-1, HSV-2, *Chlamydia trachomatis*, *Treponema pallidum*, *Haemophilus ducreyi*, and *Klebsiella granulomatis* to ensure comprehensive analysis and accurate diagnosis of GUD and associated infections. Participants were excluded from the study if they did not present with visible or unhealed genital ulcers, as the study specifically targeted individuals with clinical symptoms of GUD. Additionally, individuals who refused to provide written informed consent were not included, as consent was a prerequisite for participation. Those whose blood or swab samples were insufficient or unsuitable for laboratory testing were also excluded to ensure the integrity of the diagnostic results. Furthermore, participants with incomplete socio-demographic or clinical information were excluded, as comprehensive data were essential for the study’s analysis and conclusions.

### 2.1. Data Collection

Data for the study were obtained from a study by Tshaka et al. (2022) [4]. The clinical records were collected from May 2018 to July 2019, with inclusion criteria requiring complete demographic and clinical records. Disease test results were categorized into three groups: positive, negative, and missing data. The study recruited 105 participants due to the inclusion of additional participants who met the selection criteria for the epidemiology of GUD but were not part of the original study of etiology of GUD, resulting in a broader and more comprehensive dataset for analysis. Blood and genital ulcer samples were collected from participants to test for HIV and syphilis. Syphilis testing was performed using the Rapid Plasma Reagin (RPR) test (Fortress Diagnostics Limited, Antrim, UK), followed by the Treponema pallidum Hemagglutination Assay (TPHA) as a confirmatory test (Fortress Diagnostics Limited, Antrim, UK). HIV testing was conducted using the Onsite™ HIV 1/2 Ab Plus Combo Rapid Test (CTK Biotech, Inc., Poway, CA, USA), in accordance with the manufacturer’s instructions. DNA was extracted from genital ulcer swabs using the phenol-chloroform method (ThermoFisher Scientific, Waltham, MA, USA), also following the manufacturer’s protocol.

### 2.2. Data Processing

Categorical variables, such as sex and substance abuse, were coded for analysis, while “not done” test results were analyzed separately to identify testing gaps. Age was grouped into categories (<20, 20–29, 30–39, 40–49, 50–59, and 60+) for a more detailed examination of demographic trends.

### 2.3. Statistical Analysis

The results included descriptive statistics calculating prevalence rates for each disease across demographic and behavioral factors. Python (version 3.8) and R (version 4.1.1) software were used for data analysis. Correlation analysis, using Pearson correlation coefficients, examined relationships between diseases, age, and substance abuse. Additionally, co-occurrence analysis computed rates of simultaneous positivity for multiple diseases, providing insights into patterns of disease overlap.

## 3. Results

The demographic distribution in Table 1 reveals a predominance of young, unemployed, and rural female attendees with at least secondary education. These findings underscore the need for age- and sex-sensitive STI interventions, particularly targeting rural and unemployed populations who may face structural barriers to accessing timely diagnosis and treatment.

Table 2 presents key behavioral factors associated with GUD and STIs among the 105 participants. These include substance use and the number of sexual partners, which are well-established risk factors for STI transmission.

### 3.1. Sex-Based Prevalence

The analysis of sex-specific disease distribution among clinic attendees revealed distinct patterns. Female attendees exhibited a higher prevalence of HIV (47.00%) compared to male attendees (22.00%), with male attendees showing relatively higher negative test rates, underscoring sex-specific differences in exposure and testing outcomes. For syphilis (Rapid Plasma Reagin (RPR) results), both sexes predominantly tested negative, female attendees at 5.00% and male attendees at 3.00%. Negative results were also common for HSV-1 and HSV-2 (Herpes Simplex Viruses 1 and 2), though female attendees had marginally higher testing rates. Similarly, Chlamydia trachomatis testing showed a predominance of negative results with minimal positive cases, yet female attendees were tested more frequently, albeit with persistent disparities in coverage. For Treponema pallidum, *Haemophilus ducreyi*, and *Klebsiella granulomatis*, the results were largely negative or with missing data, with female attendees showing slightly higher rates of “missing data” entries, highlighting gaps in comprehensive testing across both sexes. These findings emphasize the need for targeted interventions to address sex-specific disparities in disease prevalence and diagnostic coverage. The prevalence of positive results for HIV and syphilis was notably higher among female attendees, highlighting a potential sex disparity in exposure or susceptibility to these conditions.

### 3.2. Age Trends

The analysis revealed age-specific trends in disease prevalence and testing coverage. HIV prevalence was highest in the 30–49 age group, with rates peaking at 66.67% in those aged 30–39 and 76.47% in the 40–49 age group. Syphilis prevalence was most pronounced in the 30–39 age group (12.12%), while younger individuals (<20 and 20–29) demonstrated elevated rates of HSV-2 and *Chlamydia trachomatis*.

**Figure 1:**
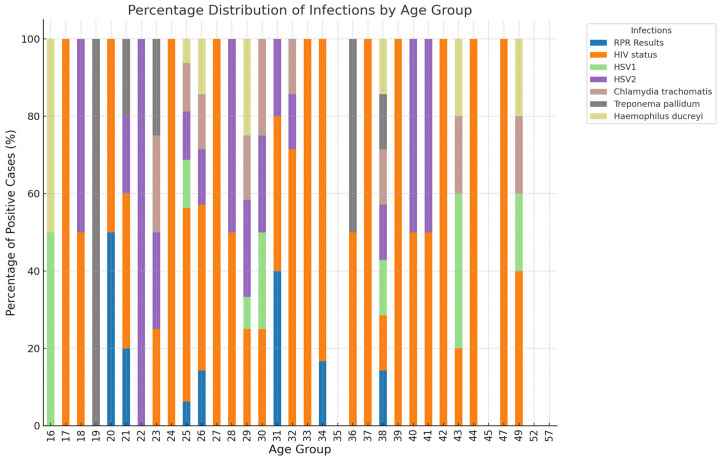
Distribution of GUD by age group.

Figure 1 indicates that HIV is the most prominent infection across almost all age groups, reflecting a widespread burden of the disease. The percentage of HIV-positive cases appears to increase with age, suggesting prolonged exposure or cumulative risk factors, such as multiple sexual partners and a lack of early intervention. In contrast, younger individuals, particularly those in their 20s and 30s, exhibit a higher proportion of HSV-2 and *Chlamydia trachomatis*, both of which are common STIs. This suggests that younger populations may engage in riskier sexual behaviors, such as unprotected sex and multiple partners, increasing their vulnerability to these infections. Syphilis, as indicated by RPR results, shows a relatively low burden across all age groups, suggesting either effective screening and treatment efforts or underdiagnosis in some cases. Furthermore, *Haemophilus ducreyi* appears in minimal proportions, while *Klebsiella granulomatis* was not detected in any of the age groups. This suggests that these pathogens are not major contributors to GUD within this population, consistent with the declining prevalence of chancroid and donovanosis reported in other studies. These results highlight age-related variations in STI distribution, with younger populations more affected by HSV-2 and *Chlamydia trachomatis*, while HIV remains a significant concern across all age groups.

### 3.3. Infection Trends by Age

The analysis of disease testing across age groups revealed consistent patterns. Negative RPR results dominated all age groups, with few positive cases; however, “missing data” results were more common among older individuals, highlighting gaps in testing. Similarly, negative results for HSV-1 and HSV-2 were predominant, particularly in younger cohorts, while older age groups had more incomplete testing. *Chlamydia trachomatis* testing showed consistent negative results across age groups, with minimal positive cases, yet testing gaps mirrored those of HSV, particularly in older populations. Older age groups exhibited higher rates of unperformed tests, indicating inconsistencies in diagnostic efforts for GUD. Key trends emerged, with the 20–39 age group showing a higher prevalence of positive HIV results, marking it as a priority for targeted interventions. Conversely, older age groups (50+) demonstrated significant testing gaps for multiple diseases, underscoring the need for improved diagnostic coverage in this demographic. Our study also enrolled a few participants in this group.

The analysis of disease prevalence across age groups highlighted distinct patterns. HIV prevalence peaked in the 40–49 age group (76.47%), followed by the 30–39 age group (66.67%), with a noted prevalence in the 20–29 age group (37.50%). Syphilis prevalence, measured through RPR results, was highest in the 30–39 age group (12.12%), with no positive cases reported in individuals aged <20, 50–59, or 60+ years. For HSV-1 and HSV-2, HSV-1 prevalence peaked in the 40–49 age group (17.65%), with notable rates in those aged <20 (10.00%) and 30–39 (6.06%) years. HSV-2 showed steady prevalence across younger and middle age groups, peaking in the 20–29 cohort (15.63%). *Chlamydia trachomatis* exhibited higher prevalence in the 40–49 (11.76%) and 20–29 (9.38%) age groups. Treponema pallidum and *Haemophilus ducreyi* were generally rare but appeared more frequently in younger and middle-aged adults. *Klebsiella granulomatis* showed no detected cases across any age group. These findings underscore age-specific disease trends, with younger and middle-aged groups being more affected, and the need for targeted health interventions across these demographics.

### 3.4. Occupation-Based Prevalence

The analysis of GUD patterns by occupation revealed notable trends. HIV prevalence was highest among employed individuals (65.91%), potentially linked to mobility or workplace exposure, followed by unemployed individuals (50.98%), reflecting socioeconomic vulnerabilities. Students had a lower prevalence (17.24%), likely due to younger age demographics. *Chlamydia trachomatis* showed a significantly higher prevalence among unemployed individuals (17.65%), highlighting a critical need for targeted interventions in this group. For syphilis, unemployed individuals had the highest prevalence (7.84%), while employed and student groups exhibited moderate rates (~4.50–6.90%). HSV-1 and HSV-2 were more prevalent among unemployed individuals (HSV-1: 11.76%, HSV-2: 19.61%), marking this group as particularly at risk, whereas students and employed individuals showed lower rates. *Treponema pallidum* was more frequent among students (17.24%), and *Haemophilus ducreyi* demonstrated a unique 100% prevalence in the self-employed group, though this may be skewed by a limited sample size. These findings emphasize the occupational disparities in disease prevalence, with unemployed individuals consistently showing higher rates for multiple conditions, signaling a need for focused public health interventions tailored to at-risk occupational groups.

**Figure 2:**
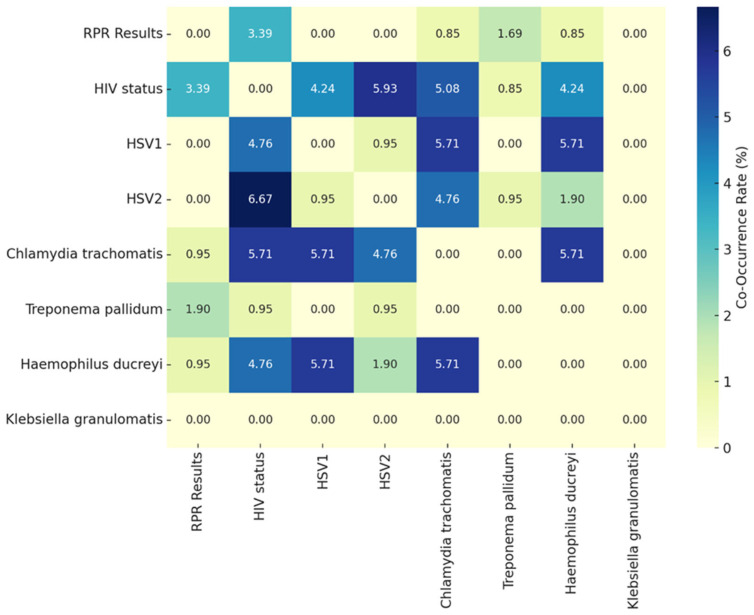
Co-occurrence and correlation rates among genital ulcer diseases.

The analysis of co-occurrence and correlation rates among diseases in Figure 2 revealed significant patterns. The intensity of each color cell represents the proportion (%) of participants who tested positive for both conditions simultaneously. Darker shades indicate a higher rate of co-occurrence, while lighter shades represent lower or no overlap between the diseases. High co-occurrence was observed for HIV and HSV-2 (6.67%), highlighting shared risk factors or overlapping populations, while *Chlamydia trachomatis* showed notable overlap with HSV-1 (5.71%) and HSV-2 (4.76%), likely due to common transmission routes. Similarly, *Haemophilus ducreyi* co-occurred with HSV-1 *and Chlamydia trachomatis* (5.71%), indicating potential co-infection patterns. Low or zero co-occurrence rates were notable for RPR results, which rarely overlapped with other diseases, and *Klebsiella granulomatis*, which showed no co-occurrence across all conditions in the dataset. Correlation analysis revealed strong links between HSV-1 and *Haemophilus ducreyi* (0.64) and between *Chlamydia trachomatis* and HSV-1 (0.56), suggesting shared risk factors. Conversely, weak or negative correlations were found for RPR results and HSV2 (−0.13), HIV status, and *Treponema pallidum* (−0.19), indicating limited overlap between these conditions. Minimal relationships were observed for diseases like HIV and syphilis (RPR results), reflecting low co-dependence. These findings underscore the need for integrated diagnostics for diseases with high co-occurrence, such as HIV and genital herpes, and targeted prevention strategies addressing common pathways for infections *Chlamydia trachomatis* and HSV-1/HSV-2. Additionally, the lack of overlap in some diseases highlights the importance of investigating testing gaps and population-specific risks to refine public health interventions.

**Figure 3:**
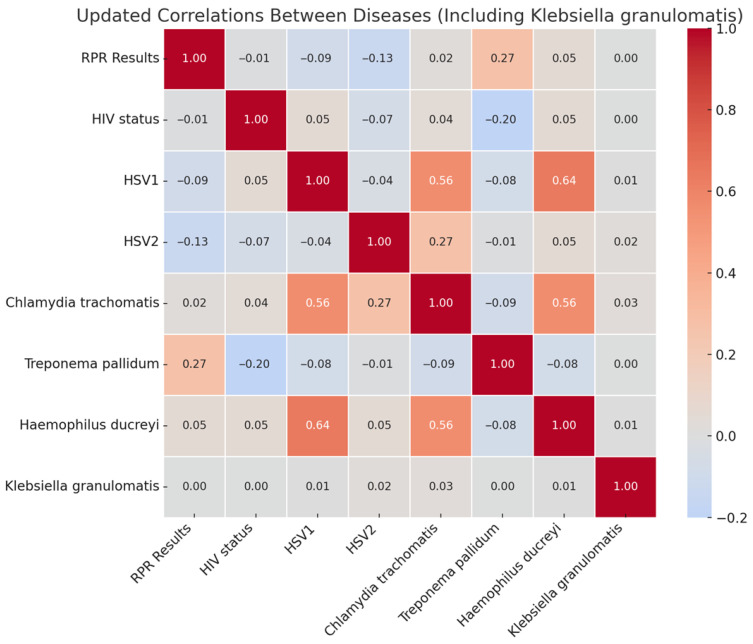
Correlation patterns and co-occurrence of sexually transmitted infections.

The correlation analysis of STIs in Figure 3 revealed key patterns of disease co-occurrence and independence. The color gradient (from deep red to deep blue) corresponds to the Pearson correlation coefficients between STI pathogens. Positive correlations (closer to +1) are shown in red shades, negative correlations (closer to −1) in blue shades, and neutral/no correlation (around 0) appear in white or light tones. Strong positive correlations were observed between HSV-1 and *Haemophilus ducreyi* (0.64), *Chlamydia trachomatis* and HSV-1 (0.56), and *Chlamydia trachomatis* and *Haemophilus ducreyi* (0.56), suggesting shared transmission routes and behavioral risk factors such as unprotected sexual contact. Additionally, HSV-2 and *Chlamydia trachomatis* (0.27) showed a moderate correlation, indicating potential overlap due to common transmission pathways. Conversely, weak or negative correlations were noted between HIV status and *Treponema pallidum* (−0.20) and RPR results and HSV2 (−0.13), indicating that these infections do not frequently co-occur, possibly due to differing risk factors, testing interventions, or distinct transmission dynamics. Minimal relationships were found between HIV status and other STIs, as well as *Treponema pallidum* and other diseases, suggesting these infections often occur independently. *Klebsiella granulomatis* exhibited nearly zero correlation with all other infections, reinforcing its rarity in this study population and the distinct epidemiology of donovanosis. These findings underscore the importance of integrated diagnostic strategies for strongly correlated infections while emphasizing the need for targeted screening and prevention efforts for infections with independent transmission patterns.

## 4. Discussion

The observed sex disparities in HIV prevalence and testing outcomes highlight significant public health concerns. Female attendees exhibited a notably higher prevalence of HIV (47.00%) compared to male attendees (22.00%), which aligns with findings that indicate women often face unique vulnerabilities that increase their risk of HIV infection. Factors contributing to this heightened risk include social determinants such as intimate partner violence, economic instability, and limited access to healthcare services, which disproportionately affect women [14,15]. Furthermore, the stigma surrounding HIV can deter individuals from seeking testing, with studies indicating that women may have more access to testing through antenatal care services, thereby influencing the observed prevalence rates [15].

Regarding testing outcomes, male attendees demonstrated relatively higher negative test rates, suggesting that they may be less likely to engage in testing behaviors than female attendees. This aligns with research indicating that men, particularly in certain cultural contexts, may avoid testing due to stigma or perceived masculinity norms that discourage vulnerability [16,17]. The slight difference in syphilis positivity rates, with female attendees at 5.00% compared to male attendees at 3.00%, further underscores the need for sex-specific approaches to sexual health education and testing initiatives [18].

To improve male participation in STI testing and reduce the burden of co-infections such as HIV and HSV-2, a multifaceted public health approach is required. Key strategies include implementing male-centered health campaigns that use culturally appropriate and stigma-free messaging, offering workplace-based mobile testing in male-dominated environments such as construction sites and mines, and integrating STI screening into routine male healthcare visits. Additionally, partner notification and index testing should be promoted, while digital platforms such as SMS and WhatsApp can be leveraged to engage younger male populations and increase testing uptake. To prevent STI co-infections, comprehensive sexual health education must be provided to address high-risk behaviors, alongside the distribution of prevention packages—including condoms, pre-exposure prophylaxis (PrEP), and routine STI screening—for vulnerable groups. Health systems should also strengthen both syndromic and laboratory-based diagnostic capacities, implement vaccination programs like HPV for both men and women, and enforce effective partner treatment and contact tracing protocols. Together, these interventions can enhance early diagnosis, reduce transmission rates, and improve sexual health outcomes across diverse populations.

The sex-specific differences in HIV prevalence and testing outcomes necessitate tailored public health strategies that address the unique challenges faced by different populations. Efforts should focus on increasing awareness, reducing stigma, and improving access to testing services, particularly for women and marginalized groups, to enhance overall health outcomes and reduce the incidence of HIV.

The prevalence of negative test results for STIs such as HIV, herpes simplex virus (HSV-1 and HSV-2), and *Chlamydia trachomatis*, alongside the higher testing rates among female attendees, underscores the complexities of STI screening and public health strategies. In the context of herpes simplex virus (HSV), both HSV-1 and HSV-2 testing results predominantly reflect negative outcomes, although female attendees tend to have slightly higher testing rates. This trend may be attributed to increased awareness and proactive health-seeking behaviors among women, particularly in reproductive health contexts where routine screenings are more common [19].

*Chlamydia trachomatis* testing also reveals a predominance of negative results, with a significant proportion of positive cases reported among high-risk populations. Studies indicate that while the infection can often be asymptomatic, the overall testing rates among female attendees are higher, reflecting a targeted approach to screening in populations at risk [20,21]. The literature suggests that age and socioeconomic factors significantly influence the prevalence of *Chlamydia* infections, particularly among younger women aged 20–24, who are at greater risk due to various behavioral and biological factors [22,23]. Furthermore, disparities in access to testing services continue to persist, highlighting the need for improved outreach and education efforts to ensure equitable coverage across different demographics [24].

In addition to sex and age disparities, geographic location may also influence STI testing behavior and infection prevalence. Although our study included a relatively balanced distribution of participants from rural (58%) and urban (42%) settings, structural and social determinants related to residence likely impacted health-seeking behavior. Rural residence may be associated with delayed care, reduced access to diagnostic services, and lower awareness of STI risks, all of which can contribute to underdiagnosis and increased burden of co-infections. Conversely, urban participants may benefit from better access to clinics and information. While the current analysis did not statistically disaggregate results by residence, we acknowledge that place of residence may have influenced the observed trends and recommend this as an area for further research to explore potential geographic disparities in STI outcomes. The genital ulcer disease is primarily caused by STIs, notably syphilis, caused by *Treponema pallidum*, and genital herpes, caused by herpes simplex virus type 2. These two pathogens represent the leading etiologies of GUD in sub-Saharan Africa, including South Africa, and were the focus of our diagnostic analysis. Moreover, HIV prevalence varies significantly across South Africa’s provinces and districts. The Eastern Cape, particularly the O.R. Tambo District, where this study was conducted, has consistently reported some of the highest HIV rates nationally. This regional variation is shaped by factors such as socioeconomic inequalities, migration dynamics, and healthcare access disparities.

For other pathogens such as *Treponema pallidum* (causing syphilis), *Haemophilus ducreyi* (causing chancroid), and *Klebsiella granulomatis* (causing donovanosis), the results are largely negative, indicating low prevalence in the tested populations. The negative results for *Treponema pallidum*, in particular, are significant as they reflect the effectiveness of public health interventions aimed at reducing syphilis transmission rates. While negative test results for STIs are common across various pathogens, the higher testing rates among female attendees highlight the importance of sex-specific health strategies. Continued efforts to enhance testing accessibility and education, particularly for high-risk populations, are essential in addressing the disparities in STI prevalence and ensuring comprehensive sexual health services.

The analysis of age-specific trends in disease prevalence and testing coverage reveals significant disparities in the rates of HIV, *Treponema pallidum*, HSV-2, and *Chlamydia trachomatis* across different age groups. Notably, HIV prevalence peaks in the 30–49 age group, with alarming rates reported in various studies, although specific figures such as 66.67% among those aged 30–39 and 76.47% in the 40–49 age group require further verification. The importance of targeted interventions for these age groups to achieve the 95-95–95 targets for HIV treatment and care is emphasized in the literature, including findings from Marinda et al., who reported high awareness and treatment rates among HIV-positive individuals [25]. Furthermore, the prevalence of syphilis is most pronounced in the 30–39 age group, where rates have been reported to be significant, indicating a need for enhanced screening and treatment protocols for STIs in this demographic [25]. In contrast, younger individuals, particularly those under 30, exhibit elevated rates of HSV2 and *Chlamydia trachomatis*, suggesting a different pattern of sexual health risks that necessitates age-appropriate educational and preventive measures [25]. The findings align with studies indicating that younger populations often engage in riskier sexual behaviors, leading to higher STI rates [25].

A critical concern highlighted in the analysis is the significant testing gaps observed in older age groups (50+), underscoring the urgent need for improved diagnostic coverage in this demographic. Hlongwane and Madiba emphasize that healthcare providers often overlook the HIV risk among older adults, leading to late diagnoses and advanced disease stages at the time of detection [26]. This is further supported by research from Kiplagat et al., which illustrates the dual stigma faced by older adults living with HIV, complicating their access to care and testing services [27]. The lack of awareness and targeted outreach for older populations contributes to these gaps, as many older adults do not perceive themselves as at risk for HIV, leading to underutilization of testing services and detection [26,27].

Moreover, the COVID-19 pandemic has exacerbated these issues, as testing rates have declined significantly due to healthcare disruptions, particularly affecting older adults who are already at higher risk for severe outcomes from both HIV and COVID-19 [28,29]. The need for comprehensive strategies to enhance testing and treatment access for older adults is critical, as indicated by the findings of Gebremeskel et al., which highlight the disparities in HIV testing uptake among older adults in Sub-Saharan Africa [30].

The analysis of HIV status across different age groups reveals significant trends in prevalence and testing outcomes, particularly highlighting the high rates of positive results in the 20–29 and 30–39 age groups. This observation aligns with findings from Vandormael et al. (2019), who documented a notable decline in HIV incidence among older populations, suggesting that younger individuals are more frequently diagnosed with HIV due to higher exposure risks and behavioral factors [31]. The systematic review by Frank et al. further supports this by providing a comprehensive overview of global HIV trends, indicating that younger age groups consistently show higher prevalence rates compared to older cohorts [32].

In contrast, negative HIV test results appear to be relatively stable across all age groups, although they are less common among both younger and older populations. This pattern suggests that, while younger individuals may engage in riskier behaviors leading to higher infection rates, older adults may also experience lower testing rates, which could contribute to the observed decline in prevalence in these age groups [33]. The consistency of negative results across age groups may reflect a broader trend of increased awareness and preventive measures among the general population, as noted by Birdthistle et al., who highlighted the importance of targeted interventions to reduce HIV incidence among young women [34].

Moreover, the age-related variations in HIV exposure and testing outcomes can be attributed to several factors, including social stigma, access to healthcare, and the effectiveness of public health campaigns. For instance, Haeuser et al. pointed out that local shifts in HIV prevalence are influenced by demographic changes and the scale-up of antiretroviral therapy (ART), which has been more effectively implemented in younger populations [35]. This is corroborated by the findings of Akullian et al., who noted significant age shifts in HIV incidence patterns, indicating that younger cohorts are more affected by the epidemic [36].

The implications of these trends are critical for public health strategies aimed at improving testing and treatment access. As highlighted by Patel et al., there is a pressing need to enhance HIV testing among younger populations while also addressing the barriers that older adults face in accessing care [37]. The literature suggests that comprehensive approaches, including education and outreach tailored to specific age groups, are essential for reducing HIV prevalence and improving health outcomes across all demographics [38,39].

The analysis of disease testing across various age groups reveals notable patterns in the prevalence of STIs and the associated testing outcomes. Negative results for the RPR test for syphilis were predominant across all age groups, indicating a low prevalence of positive cases. However, a concerning trend emerged with “Not done” results being more prevalent among older individuals, suggesting significant gaps in testing coverage in this demographic [40,41,42]. This finding is consistent with the broader literature, which indicates that older adults often face barriers to accessing STI testing, including stigma and healthcare provider biases [43]. Moreover, *Klebsiella granulomatis* yielded negative results, with the data suggesting that, while younger and middle-aged groups are more frequently tested and diagnosed with certain STIs, older populations face significant barriers to adequate testing, which may contribute to undiagnosed cases and ongoing transmission within these age groups [44].

A critical observation from the analysis is the elevated prevalence of positive HIV results in the 20–39 age group, marking this cohort as a priority for targeted health interventions. Specifically, the 40–49 age group exhibited the highest HIV prevalence at 76.47%, followed closely by the 30–39 age group at 66.67%, with a significant presence in the 20–29 group at 37.50% [45,46]. Conversely, older age groups (50+) displayed significant gaps in testing across multiple diseases, underscoring the urgent need for improved diagnostic coverage and health service accessibility for this demographic [44,47]. The disparities in testing practices and disease prevalence across age groups highlight a critical area for public health initiatives aimed at reducing STI transmission and improving overall health outcomes [43].

The analysis of disease patterns by occupation reveals significant disparities in the prevalence of STIs across different employment statuses. The findings indicate that HIV prevalence is notably highest among employed individuals, reported at 65.91%. This elevated rate may be attributed to factors such as increased mobility, workplace exposure, and potentially higher engagement in risk-taking behaviors associated with occupational environments [42,43]. In contrast, the prevalence among unemployed individuals stands at 50.98%, which reflects the socioeconomic vulnerabilities that often accompany unemployment, including limited access to healthcare services and education regarding safe practices [46]. Students, representing a younger demographic, exhibited a significantly lower HIV prevalence of 17.24%, likely due to their age and possibly lower engagement in high-risk behaviors [48].

*Chlamydia trachomatis* prevalence presents a critical concern, particularly among unemployed individuals, where it reaches 17.65%. This statistic underscores the urgent need for targeted public health interventions for this group, as they may lack access to preventive healthcare and education [44]. The prevalence of syphilis also mirrors this trend, with unemployed individuals reporting the highest rates at 7.84%. In comparison, employed individuals and students exhibited moderate rates of syphilis, ranging from approximately 4.5% to 6.9% [45].

HSV-1 and HSV-2 further illustrate the occupational disparities in disease prevalence. Unemployed individuals demonstrated higher rates of HSV-1 (11.76%) and HSV-2 (19.61%), indicating that this group is particularly at risk for these infections. Conversely, students and employed individuals reported lower prevalence rates, suggesting a potential correlation between employment status and exposure risk [47].

Interestingly, *Treponema pallidum*, the causative agent of syphilis, was found to be more prevalent among students at 17.24%. This finding may reflect specific behavioral patterns within this demographic, such as increased sexual activity or lack of awareness regarding STI risks. Additionally, *Haemophilus ducreyi*, responsible for chancroid, exhibited a striking 100% prevalence in the self-employed group; however, this statistic may be skewed due to a limited sample size, necessitating caution in interpretation.

The analysis of co-occurrence and correlation rates among various STIs reveals significant patterns that underscore the interconnectedness of these diseases. The data indicate a high co-occurrence rate between HIV and HSV-2, recorded at 6.67%. This finding suggests shared risk factors or overlapping populations, which is consistent with the existing literature that highlights the role of HSV-2 as a significant risk factor for HIV acquisition [49,50]. The prevalence of HSV-2 infection is known to disrupt the genital mucosa, thereby providing a portal for HIV entry, which may explain the observed co-occurrence [51,52].

*Chlamydia trachomatis* also exhibited notable overlap with HSV-1 and HSV-2, likely due to common transmission routes and risk behaviors associated with these infections [53,54]. The co-occurrence of *Haemophilus ducreyi* with both HSV-1 and *Chlamydia trachomatis* further indicates potential co-infection patterns that warrant attention in public health strategies [55]. The presence of multiple infections within the same population can complicate treatment and prevention efforts, highlighting the need for integrated diagnostic approaches.

Conversely, low or zero co-occurrence rates were observed for RPR test results for syphilis, which rarely overlapped with other diseases. Similarly, *Klebsiella granulomatis* showed no co-occurrence across all conditions in the dataset, suggesting that these infections may have distinct epidemiological profiles that do not frequently intersect [56]. This lack of overlap emphasizes the importance of targeted screening and prevention strategies for these specific infections.

Correlation analysis further elucidates the relationships between these diseases. Strong correlations were identified between HSV-1 and *Haemophilus ducreyi* and between *Chlamydia trachomatis* and HSV-1, indicating shared risk factors and potential pathways for transmission [57]. In contrast, weak or negative correlations were found for RPR results and HSV-2 and between HIV status and *Treponema pallidum*, suggesting limited overlap between these conditions [58]. The minimal relationships observed for diseases like HIV and RPR results reflect low co-dependence, indicating that interventions targeting one may not necessarily impact the other.

The analysis of disease correlations within the context of STIs reveals distinct patterns that can inform public health strategies and clinical practices. Strong correlations were identified between HSV-1 and *Haemophilus ducreyi* (correlation coefficient of 0.64), suggesting a potential for co-occurrence or shared risk factors between these two infections [59]. This correlation may be attributed to overlapping transmission routes, as both infections are often associated with sexual activity and can occur in similar populations.

Additionally, a significant correlation was observed between *Chlamydia trachomatis* and HSV-1 (0.56), further indicating a link likely driven by common transmission pathways [59,60]. The presence of *Chlamydia trachomatis*, a prevalent bacterial STI, may increase susceptibility to HSV-1 infection due to mucosal disruption during sexual contact. This finding highlights the importance of considering multiple infections in the same patient population, as co-infections can complicate treatment and management strategies.

Conversely, weak or negative correlations were noted between RPR results and HSV-2 (−0.13), reflecting minimal overlap between these two conditions. This suggests that individuals who test positive for one may not necessarily be at increased risk for the other, indicating distinct epidemiological profiles [61,62]. Similarly, the correlation between HIV status and *Treponema pallidum* (−0.19) suggests a lower likelihood of concurrent infections in the dataset, which may be influenced by differing risk factors and transmission dynamics associated with each infection [63]. Moreover, several diseases, such as HIV and RPR results, demonstrated minimal or negligible correlations with other conditions, indicating limited interdependence. This lack of correlation underscores the necessity for tailored public health interventions that address the unique characteristics and risk factors associated with each STI [64,65].

A key limitation of this study is the potential for selection bias, as the sample was restricted to symptomatic patients attending public clinics in Mthatha. This group may not be representative of the broader regional population, particularly those who are asymptomatic, do not seek care, or access alternative healthcare providers. Due to the lack of a direct comparison with regional population-level data, the generalizability of the findings may be limited. Future studies incorporating community-based sampling or district-level surveillance comparisons are recommended to address this gap. As a result, the representativeness of the epidemiological results cannot be reliably estimated. The observed trends may disproportionately reflect individuals with more severe or persistent symptoms who are more likely to present at clinics. This limitation restricts broader inference and highlights the need for future population-based studies to accurately characterize the regional burden and distribution of GUD and STI co-infections.

## 5. Conclusions

The analysis highlights the intricate interplay of age-specific trends in disease prevalence and testing coverage, revealing critical implications for public health strategies. It underscores the necessity of targeted interventions to address the unique needs and barriers faced by different demographics, particularly older adults, while improving testing rates and diagnostic coverage for all age groups. The higher rates of positive results among younger individuals, alongside stable negative outcomes across age groups, suggest complex factors influencing exposure and testing behaviors. These findings emphasize the importance of integrated diagnostics for co-occurring infections, such as HIV and HSV-2, and tailored prevention strategies for diseases with shared transmission pathways, like *Chlamydia trachomatis* and HSV-1/HSV-2. Furthermore, the occupational disparities in disease prevalence, particularly among unemployed individuals, signal an urgent need for focused outreach, education, and testing programs targeting at-risk populations. Investigating testing gaps and population-specific risks for infections with low correlation remains vital for developing effective interventions. Regular screening for both GUD and HIV is a cornerstone of effective public health response, especially in high-prevalence regions such as the Eastern Cape. Integrating routine screening into primary care and community-based services can improve early detection, limit disease progression, and reduce transmission. Strengthening access to screening and diagnostic services will be essential to closing existing gaps and achieving national targets for STI and HIV control. Overall, the results call for comprehensive, demographically informed public health efforts to enhance health outcomes and reduce transmission rates across diverse populations.

## Figures and Tables

**Table 1:** Demographic characteristics of study participants.

Characteristic	Category	Frequency (*n*)
Sex	Female	85
Sex	Male	20
Age Group	<20	20
Age Group	20–29	32
Age Group	30–39	33
Age Group	40–49	17
Age Group	50–59	2
Age Group	60+	1
Residence	Urban	44
Residence	Rural	61
Occupation	Employed	22
Occupation	Unemployed	51
Occupation	Student	29
Occupation	Self-employed	3
Education Level	No formal education	7
Education Level	Primary	21
Education Level	Secondary	45
Education Level	Tertiary	32

**Table 2:** Behavioral characteristics of study participants.

Behavioral Factor	Category	Frequency (*n*)
Substance Use	Yes	38
Substance Use	No	67
Sexual Partners	1	44
Sexual Partners	2–3	39
Sexual Partners	4 or more	22

## Data Availability

Data can be requested from the corresponding author.

## References

[1] Zheng Y., Yu Q., Lin Y., Zhou Y., Lan L., Yang S., Wu J. (2022). Global burden and trends of sexually transmitted infections from 1990 to 2019: An observational trend study. Lancet Infect. Dis..

[2] Huyveneers L.E., Maphanga M., Umunnakwe C.N., Bosman-de Boer L., Moraba R.S., Tempelman H.A., Wensing A.M., Hermans L.E. (2023). Prevalence, incidence, and recurrence of sexually transmitted infections in HIV-negative adult women in a rural South African setting. Trop. Med. Int. Health.

[3] Dhar C.P., Feuerstein J.L., Salas-Humara C. (2024). Overview of Vaginal Ulcerative Disease. Pediatr. Ann..

[4] Tshaka T.R., Singh R., Apalata T.R., Mbulawa Z.Z.A. (2022). Aetiology of genital ulcer disease and associated factors among Mthatha public clinic attendees. S. Afr. J. Infect. Dis..

[5] Musonda P., Halwiindi H., Kaonga P., Ngoma-Hazemba A., Simpungwe M., Mweemba M., Tembo C., Zyambo C., Chisoso J., Munakampe M. (2024). HIV, syphilis and sexual-risk behaviours’ prevalence among in-and out-of-school adolescent girls and young women in Zambia: A cross-sectional survey study. PLoS ONE.

[6] Korkusuz R., Şenoğlu S. (2020). Syphilis Seroprevalence and Associated Risk Factors in HIV-infected Individuals. Mediterr. J. Infect. Microbes Antimicrob..

[7] Gilbert L., Dear N., Esber A., Iroezindu M., Bahemana E., Kibuuka H., Owuoth J., Maswai J., Crowell T.A., Polyak C.S. (2021). Prevalence and risk factors associated with HIV and syphilis co-infection in the African Cohort Study: A cross-sectional study. BMC Infect. Dis..

[8] Kularatne R.S., Muller E.E., Maseko D.V., Kufa-Chakezha T., Lewis D.A. (2018). Trends in the relative prevalence of genital ulcer disease pathogens and association with HIV infection in Johannesburg, South Africa, 2007–2015. PLoS ONE.

[9] Abdallah I., Armstrong-Mensah E., Alema-Mensah E., Cheryl J. (2017). Demographic and behavioural risk factors associated with Trichomonas vaginalis among South African HIV-positive men with genital ulcer disease: A cross-sectional study. BMJ Open.

[10] Smith J.S., Robinson N.J. (2002). Age-specific prevalence of infection with herpes simplex virus types 2 and 1: A global review. J. Infect. Dis..

[11] Wand H., Reddy T., Dassaye R., Moodley J., Naidoo S., Ramjee G. (2020). Estimating prevalence and incidence of sexually transmitted infections among South African women: Implications of combined impacts of risk factors. Int. J. STD AIDS.

[12] Mathias B.A., Muñoz T.I., Guaicha E.P., Quinde A.P., Herrera M.F. (2024). Side effects of Sexually Transmitted Infections (STIs) on female fertility: Diagnostic and treatment strategies–a literature review. Ibero-Am. J. Health Sci. Res..

[13] Kojima N., Klausner J.D. (2018). An Update on the Global Epidemiology of Syphilis. Curr. Epidemiol. Rep..

[14] Malama K., Logie C.H., Sokolovic N., Skeritt L., O’Brien N., Cardinal C., Gagnier B., Loutfy M., Kaida A., de Pokomandy A. (2023). Pathways from HIV-related stigma, racial discrimination, and gender discrimination to HIV treatment outcomes among women living with HIV in Canada: Longitudinal cohort findings. JAIDS J. Acquir. Immune Defic. Syndr..

[15] Ha J.H., Van Lith L.M., Mallalieu E.C., Chidassicua J., Pinho M.D., Devos P., Wirtz A.L. (2019). Gendered relationship between HIV stigma and HIV testing among men and women in Mozambique: A cross-sectional study to inform a stigma reduction and male-targeted HIV testing intervention. BMJ Open.

[16] Smith P.J., Davey D.J., Green H., Cornell M., Bekker L.G. (2021). Reaching underserved South Africans with integrated chronic disease screening and mobile HIV counselling and testing: A retrospective, longitudinal study conducted in Cape Town. PLoS ONE.

[17] Turan J.M., Elafros M.A., Logie C.H., Banik S., Turan B., Crockett K.B., Pescosolido B., Murray S.M. (2019). Challenges and opportunities in examining and addressing intersectional stigma and health. BMC Med..

[18] Klein P.W., Psihopaidas D., Xavier J., Cohen S.M. (2020). HIV-related outcome disparities between transgender women living with HIV and cisgender people living with HIV served by the Health Resources and Services Administration’s Ryan White HIV/AIDS Program: A retrospective study. PLoS Med..

[19] Naeem A., Waseem H., Ali S., Usman J., Hanif F., Furqan W. (2023). Chlamydia trachomatis infection in pelvic inflammatory disease patients–a snap shot. Infect. Dis. J. Pak..

[20] Chen H., Luo L., Wen Y., He B., Ling H., Shui J., He P., Hou X., Tang S., Li Z. (2020). Chlamydia trachomatis and human papillomavirus infection in women from southern Hunan Province in China: A large observational study. Front. Microbiol..

[21] Li T., Liu Z., Zhang D., Liao Q., Fan S., Hao M., Hong Y., Huang X., Wang H., Xiong Z. (2023). Prevalence of and risk factors for chlamydia in female outpatients with genital tract infections: A nationwide multi-center, cross-sectional study in China. Front. Public Health.

[22] Yan R.L., Ye Y.F., Fan Q.Y., Huang Y.H., Wen G.C., Li L.M., Cai Y.M., Feng T.J., Huang Z.M. (2019). Chlamydia trachomatis infection among patients attending sexual and reproductive health clinics: A cross-sectional study in Bao’an district, Shenzhen, China. PLoS ONE.

[23] Dombrowski J.C., Wierzbicki M.R., Newman L.M., Powell J.A., Miller A., Dithmer D., Soge O.O., Mayer K.H. (2021). Doxycycline versus azithromycin for the treatment of rectal chlamydia in men who have sex with men: A randomized controlled trial. Clin. Infect. Dis..

[24] Xu X., Chow E.P., Ong J.J., Hoebe C.J., Zou Z., Hocking J.S., Fairley C.K., Zhang L. (2020). Chlamydia trachomatis transmission between the oropharynx, urethra and anorectum in men who have sex with men: A mathematical model. BMC Med..

[25] Marinda E., Simbayi L., Zuma K., Zungu N., Moyo S., Kondlo L., Jooste S., Nadol P., Igumbor E., Dietrich C. (2020). Towards achieving the 90–90–90 HIV targets: Results from the south African 2017 national HIV survey. BMC Public Health.

[26] Hlongwane N., Madiba S. (2020). Navigating life with HIV as an older adult in South African communities: A phenomenological study. Int. J. Environ. Res. Public Health.

[27] Kiplagat J., Mwangi A., Chasela C., Huschke S. (2019). Challenges with seeking HIV care services: Perspectives of older adults infected with HIV in western Kenya. BMC Public Health.

[28] Huang G., Cheng W., Xu Y., Yang J., Jiang J., Pan X., Zhou X., Jiang J., Chai C. (2024). Spatiotemporal Pattern and Its Determinants for Newly Reported HIV/AIDS Among Older Adults in Eastern China From 2004 to 2021: Retrospective Analysis Study. JMIR Public Health Surveill..

[29] Waterfield K.C., Shah G.H., Etheredge G.D., Ikhile O. (2021). Consequences of the COVID-19 crisis for persons with HIV: The impact of social determinants of health. BMC Public Health.

[30] Gebremeskel A.T., Gunawardena N., Omonaiye O., Yaya S. (2021). Sex Differences in HIV Testing among Older Adults in Sub-Saharan Africa: A Systematic Review. BioMed Res. Int..

[31] Vandormael A., Akullian A., Siedner M., de Oliveira T., Bärnighausen T., Tanser F. (2019). Declines in HIV incidence among men and women in a South African population-based cohort. Nat. Commun..

[32] Frank T.D., Carter A., Jahagirdar D., Biehl M.H., Douwes-Schultz D., Larson S.L., Arora M., Dwyer-Lindgren L., Steuben K.M., Abbastabar H. (2019). Global, regional, and national incidence, prevalence, and mortality of HIV, 1980–2017, and forecasts to 2030, for 195 countries and territories: A systematic analysis for the Global Burden of Diseases, Injuries, and Risk Factors Study 2017. Lancet HIV.

[33] Hayes R.J., Donnell D., Floyd S., Mandla N., Bwalya J., Sabapathy K., Yang B., Phiri M., Schaap A., Eshleman S.H. (2019). Effect of universal testing and treatment on HIV incidence—HPTN 071 (PopART). N. Engl. J. Med..

[34] Birdthistle I., Tanton C., Tomita A., de Graaf K., Schaffnit S.B., Tanser F., Slaymaker E. (2019). Recent levels and trends in HIV incidence rates among adolescent girls and young women in ten high-prevalence African countries: A systematic review and meta-analysis. Lancet Glob. Health.

[35] Haeuser E., Serfes A.L., Cork M.A., Yang M., Abbastabar H., Abhilash E.S., Adabi M., Adebayo O.M., Adekanmbi V., Adeyinka D.A. (2022). Mapping age-and sex-specific HIV prevalence in adults in sub-Saharan Africa, 2000–2018. BMC medicine.

[36] Akullian A., Vandormael A., Miller J.C., Bershteyn A., Wenger E., Cuadros D., Gareta D., Bärnighausen T., Herbst K., Tanser F. (2021). Large age shifts in HIV-1 incidence patterns in KwaZulu-Natal, South Africa. Proc. Natl. Acad. Sci. USA.

[37] Patel D., Johnson C.H., Krueger A., Maciak B., Belcher L., Harris N., DiNenno E.A. (2020). Trends in HIV testing among US adults, aged 18–64 years, 2011–2017. AIDS Behav..

[38] Havlir D., Lockman S., Ayles H., Larmarange J., Chamie G., Gaolathe T., Iwuji C., Fidler S., Kamya M., Floyd S. (2020). What do the Universal Test and Treat trials tell us about the path to HIV epidemic control?. J. Int. AIDS Soc..

[39] Govender R.D., Hashim M.J., Khan M.A., Mustafa H., Khan G. (2021). Global epidemiology of HIV/AIDS: A resurgence in North America and Europe. J. Epidemiol. Glob. Health.

[40] Workowski K.A. (2021). Sexually Transmitted Infections Treatment Guidelines, 2021. MMWR Recomm. Rep..

[41] Willemstein I.J., Götz H.M., Visser M., Heijne J.C. (2023). HIV and syphilis testing for women and heterosexual men aged above 25 years in the Netherlands: Possibilities for targeted testing at sexual health centres. BMJ Open.

[42] Effendi I., Rosana Y., Yasmon A., Indriatmi W. (2018). Multiplex nested polymerase chain reaction for Treponema pallidum using blood is more sensitive than using serum. Universa Med..

[43] Bristow C.C., Leon S.R., Huang E., Ramos L.B., Vargas S.K., Flores J.A., Konda K.A., Caceres C.F., Klausner J.D. (2016). Field evaluation of a dual rapid immunodiagnostic test for HIV and syphilis infection in Peru. Sex. Transm. Dis..

[44] Mosen D.M., Banegas M.P., Dickerson J.F., Fellows J.L., Pihlstrom D.J., Kershah H.M., Scott J.L., Keast E.M. (2021). Evaluating the Effectiveness of Medical–Dental Integration to Close Preventive and Disease Management Care Gaps. Front. Dent. Med..

[45] Song Y., Liu Y.S., Talarico F., Zhang Y., Hayward J., Wang M., Stroulia E., Dixon R.A., Greiner R., Li X. (2023). Associations between differential aging and lifestyle, environment, current, and future health conditions: Findings from Canadian Longitudinal Study on Aging. Gerontology.

[46] Nkansah C., Serwaa D., Osei-Boakye F., Owusu-Ampomah R. (2022). Magnitude and trend of HIV and Treponema pallidum infections among blood donors in Offinso-North District, Ghana: A nine-year retrospective, cross-sectional study. Afr. Health Sci..

[47] Liu W.S., You J., Ge Y.J., Wu B.S., Zhang Y., Chen S.D., Zhang Y.R., Huang S.Y., Ma L.Z., Feng J.F. (2023). Association of biological age with health outcomes and its modifiable factors. Aging Cell.

[48] Corebima B.I., Almiradani A., Sulistijono E. (2020). Importance of Serological Tests in the Diagnosis of Asymptomatic Congenital Syphilis in Neonates: A Case Report. Arch. Pediatr. Infect. Dis..

[49] Bradley J., Floyd S., Piwowar-Manning E., Laeyendecker O., Young A., Bell-Mandla N., Bwalya J., Bock P., Fidler S., Ayles H. (2018). Sexually transmitted bedfellows: Exquisite association between HIV and herpes simplex virus type 2 in 21 communities in southern Africa in the HIV prevention trials network 071 (PopART) study. J. Infect. Dis..

[50] Reinheimer C., Doerr H.W. (2012). Prevalence of herpes simplex virus type 2 in different risk groups: Thirty years after the onset of HIV. Intervirology.

[51] Barnabas R.V., Wasserheit J.N., Huang Y., Janes H., Morrow R., Fuchs J., Mark K.E., Casapia M., Mehrotra D.V., Buchbinder S.P. (2011). Impact of herpes simplex virus type 2 on HIV-1 acquisition and progression in an HIV vaccine trial (the Step study). J. Acquir. Immune. Defic. Syndr..

[52] Sunur S., Purwoko I.H., Yahya Y.F., Pamudji R. (2021). Genital Herpes in Human Immunodeficiency Virus Infected Patients. Biosci. Med. J. Biomed. Transl. Res..

[53] Ajani T.A., Oluwasola T.A., Anaedobe C.G., Ajani M.A., Fayemiwo S.A., Bakare R.A. (2017). Correlates of genital Chlamydial trachomatis infection in a cohort of infertile women in Ibadan, Nigeria. Int. J. Reprod. Contracep. Obs. Gynecol..

[54] Beyuo T., Oppong S.A., Samba A., Beyuo V.M. (2019). Chlamydia trachomatis infection among Ghanaian women undergoing hysterosalpingography for suspected tubal factor infertility. Int. J. Gynecol. Obstet..

[55] Shukla D., Kalyan R.K., Gupta P., Venkatesh V., Agarwal A. (2024). The socio-demographic profile and clinical correlation of Chlamydia trachomatis among infertile women at a tertiary care center in North India. Int. J. Reprod. Contracept. Obstet. Gynecol..

[56] Lautenschlager S., Kemp M., Christensen J.J., Mayans M.V., Moi H. (2017). 2017 European guideline for the management of chancroid. Int. J. STD AIDS.

[57] Martinelli E., Tharinger H., Frank I., Arthos J., Piatak Jr M., Lifson J.D., Blanchard J., Gettie A., Robbiani M. (2011). HSV-2 infection of dendritic cells amplifies a highly susceptible HIV-1 cell target. PLoS Pathog..

[58] Adejumo B.I., Oronsaye F.E., Drisu U.I., Adebowale M.O., Oke O.M., Dimkpa U., Omosor K.I., Abdulrahman O.N., Ukatu E.N., Michael E.A. (2018). The Level of CD4+ T Cell Count among Reproductive Age Women Coinfected with Human Immune Virus, Hepatitis Surface Antigen and Herpes Simplex Virus in Kogi State, Nigeria. Health.

[59] Popoola V.O., Kagaayi J., Ssekasanvu J., Ssekubugu R., Kigozi G., Ndyanabo A., Nalugoda F., Chang L.W., Lutalo T., Tobian A.A. (2022). Prevalence of untreated HIV and HIV incidence among occupational groups in Rakai, Uganda: A population-based longitudinal study, 1999–2016. medRxiv.

[60] Adegbosin A.P., Adegbosin A.E. (2022). A review of socioeconomic inequalities in HIV infections among women in Low and Middle-income countries. medRxiv.

[61] Abdulkarim S., John S., Garba T., Basason H., Balogun P., Kuye J. (2024). Perceptions of TB-HIV comorbidity among the Nomads in Adamawa State, Nigeria. BMC Public Health.

[62] Kimaro L., Adinan J., Damian D.J., Njau B. (2018). Prevalence of occupational injuries and knowledge of availability and utilization of post exposure prophylaxis among health care workers in Singida District Council, Singida Region, Tanzania. PLoS ONE.

[63] Nwalozie R., Kareem J.A., Ikpo P.E. (2024). Epidemiological Distribution of Human Immunodeficiency Virus (HIV) among Residents of Port Harcourt Metropolis in Rivers State Nigeria. Asian J. Res. Infect. Dis..

[64] Li Y., Zhang X., Huang Y., Gao L., Gao Z., He M. (2024). The prevalence of Human Immunodeficiency Virus infection among voluntary blood donors in mainland China: A systematic review and meta-analysis. J. Med. Virol..

[65] Hanke D., Freuling C.M., Fischer S., Hueffer K., Hundertmark K., Nadin-Davis S., Marston D., Fooks A.R., Bøtner A., Mettenleiter T.C. (2016). Spatio-temporal analysis of the genetic diversity of arctic rabies viruses and their reservoir hosts in Greenland. PLoS Neglected Trop. Dis..

